# Screening of Surface-Exposed Lipoproteins of *Leptospira* Involved in Modulation of Host Innate Immune Response

**DOI:** 10.3389/fmicb.2022.761670

**Published:** 2022-03-24

**Authors:** Ajay Kumar, Vivek P. Varma, Syed M. Faisal

**Affiliations:** ^1^Laboratory of Vaccine Immunology, National Institute of Animal Biotechnology, Hyderabad, India; ^2^Regional Center for Biotechnology, Faridabad, India; ^3^Graduate Studies, Manipal Academy of Higher Education, Manipal, India

**Keywords:** surface lipoproteins, *Leptospira*, pathogenesis, immune modulation, host–pathogen interaction

## Abstract

*Leptospira*, a zoonotic pathogen, is capable of causing both chronic and acute infection in a susceptible host. Surface-exposed lipoproteins play a major role in modulating the host immune response by activating the innate cells like macrophages and dendritic cells or evading complement attack and killing by phagocytes like neutrophils to favor pathogenesis and establish infection. In this study, we screened some surface-exposed lipoproteins known to be involved in pathogenesis to assess their possible role in immune modulation (innate immune activation or evasion). Surface proteins of the Len family (LenB, LenD, and LenE), Lsa30, Loa22, and Lipl21 were purified in recombinant form and then tested for their ability to activate macrophages of the different host (mouse, human, and bovine). These proteins were tested for binding with complement regulators like Factor H (FH), C4 Binding Protein (C4BP), and host protease Plasminogen (PLG) and also as nucleases to access their possible role in innate immune evasion. Our results show that, of various proteins tested, Loa22 induced strong innate activation and Lsa30 was least stimulatory, as evident from the production of pro-inflammatory cytokines (interleukin-6 and tumor necrosis factor–α) and expression of surface markers [CD80, CD86, and major histocompatibility complex class II (MHCII)]. All the tested proteins were able to bind to FH, C4BP, and PLG; however, Loa22 showed strong binding to PLG correlating to plasmin activity. All the proteins except Loa22 showed nuclease activity, albeit with a requirement of different metal ions. The nuclease activity of these proteins correlated to *in vitro* degradation of neutrophil extracellular trap (NET). In conclusion, our results indicate that these surface proteins are involved in innate immune modulation and may play a critical role in assisting the bacteria in invading and colonizing the host tissue for persistent infection.

## Introduction

Leptospirosis is a zoonotic disease caused by bacterial spirochete belonging to the genus *Leptospira*. With 1.03 million cases and 58,900 deaths per year, it has become a leading cause of morbidity and mortality, especially in tropical countries (Ko et al., 2009). Lack of early detection and unavailability of vaccines capable of inducing cross-protection against various serovars are major hurdles in the containment of infection and reducing associated pathology. Little is known about *Leptospira* pathogenesis and host immune response, which has hampered the development of an effective vaccine (Ko et al., 2009). Pathogenic bacteria including *Leptospira* upon entering a susceptible host are recognized by the innate immune system, which tries to kill the bacteria by a variety of mechanism. Pattern recognition receptors like Toll-like receptors (TLRs) and Nucleotide binding oligomerization domain (NOD) like receptors (NLRs) upon recognition induce activation of innate immune cells and subsequent induction of adaptive response (Santecchia et al., 2020). Complement system comprising of soluble proteins in blood initiates a cascade of reaction and leads to the rapid killing of the pathogen by the phagocytosis and target cell lysis (Dunkelberger and Song, 2010). Phagocytes like neutrophils may kill the bacteria by generating reactive oxygen species (ROS), cytotoxic granules, antimicrobial peptides and by generating neutrophil extracellular traps (NETs) (Teng et al., 2017). To escape from these innate defenses, pathogen may induce TLR-dependent inflammatory response to favor their pathogenesis but may also escape recognition through this protective response by antigenic variation or downregulating the expression of surface proteins. *Leptospira* likely evades recognition from TLR2 and TLR4 possibly by modulating the expression of surface proteins as mice lacking these receptors were highly susceptible to infection (Chassin et al., 2009). *Leptospira* may acquire complement regulators and host proteases to escape from complement-mediated killing. To avoid killing by phagocytes like neutrophils, they exploit their surface proteins to evade extravasation and chemotaxis, opsonization, and phagocytosis (Teng et al., 2017). They may express surface proteins as nucleases that can degrade NET once it gets entrapped, as reported in other pathogens (Thammavongsa et al., 2013; Doke et al., 2017; Storisteanu et al., 2017).

Previous studies identified several surface proteins of *Leptospira* as pro-inflammatory and capable of inducing TLR2- or TLR4-dependent activation of innate immune response (Hung et al., 2006; Yang et al., 2006; Wang et al., 2012; Faisal et al., 2016). Several surface proteins have also been shown to bind Factor H (FH), C4 Binding Protein (C4BP), host protease Plasminogen (PLG), to evade from complement-mediated killing (Fraga et al., 2016). LipL21 can modulate neutrophil function by inhibiting Myeloperoxidase (MPO) (Vieira et al., 2018). *Leptospira* can induce the formation of NET; hence, it is likely that it might express surface proteins as nucleases that can degrade NET (Scharrig et al., 2015). Although several surface proteins of *Leptospira* have been identified that are involved in activation or evasion from host innate immune response, no attempt has been made to identify those proteins involved in immune modulation (both activation and evasion). On the basis of literature review, we selected some of the surface-exposed lipoproteins like LenB, LenD, LenE, Lsa30, Loa22, and LipL21 known to be involved in pathogenesis to access their role in immune modulation (Raja and Natarajaseenivasan, 2015). *Leptospira* endostatin-like proteins (Len proteins) are a family of outer membrane lipoproteins comprising LenA, LenB, LenC, LenD, LenE, and LenF, which are widely distributed in pathogenic serovars. They have an important role in pathogenesis as they are expressed during infection and have been shown to interact with components of host extracellular matrix like laminin and fibronectin (Vieira et al., 2014). LenA and LenB have been shown to bind to FH and FHR-1 (Verma et al., 2006; Stevenson et al., 2007). Loa22 (a surface protein having an OmpA-like domain) is conserved among pathogenic serovars (Ristow et al., 2007). Loa22 is expressed during infection and is strongly recognized by sera obtained from human and bovine leptospirosis (Gamberini et al., 2005; Ghosh et al., 2018). It has been shown to bind to components of ECM-like fibronectin and collagen and has led to mediate pro-inflammatory response possibly through TLR2 recognition (Barbosa et al., 2006; Barbosa and Isaac, 2020; Hsu et al., 2021). It is an essential virulence factor as the Loa22 mutant was attenuated in virulence (Ristow et al., 2007). LipL21 is the second most abundant surface protein after LipL32 and is conserved among pathogenic serovars (Cullen et al., 2003). It is immunogenic and recognized by both immune sera from humans and hamsters infected with *Leptospira* (Cullen et al., 2003; Ratet et al., 2017). Lsa30 is a novel adhesin that is shown to bind with Extracellular matrix (ECM) components, interact with C4BP and PLG (Souza et al., 2012; Fouts et al., 2016).

In the present study, we screened these proteins to assess their possible role in innate immune modulation, i.e., the ability to activate macrophages, bind to complement regulators and host proteases and act as nucleases for their possible role in degrading NET. We cloned, expressed, and purified these proteins in recombinant form and tested their ability to activate mouse, human, and bovine macrophages. In addition, we tested the binding ability of these proteins with complement regulators (FH and C4BP) and host proteases (PLG). Further, we tested the nuclease activity of these proteins and correlated this nuclease activity with the degradation of NET *in vitro*.

## Materials and Methods

### Cell Lines and Reagents

Mouse, bovine, and human macrophage cell lines RAW264.7, BoMac, and THP-1, respectively, were initially purchased from the American Type Culture Collection (Manassas, VA, United States). Cells were cultured in specified medium [Dulbecco’s Modified Eagle Medium (DMEM) or Roswell Park Memorial Institute (RPMI) 1640, Sigma, United States] supplemented with 10% Fetal bovine serum (FBS) (Invitrogen, Carlsbad, CA, United States), penicillin (100 U/ml), and streptomycin (100 mg/ml) and maintained at 37°C, 5% CO_2_. Interleukin 6 (IL-6) and tumor necrosis factor–α (TNF-α) cytokines sandwich ELISA kits specific for human or mouse or bovine were purchased from R&D system. Peridinin chlorophyll protein-Cyanine5.5 (PerCP-CY5)–conjugated anti-MHCII, Phycoerythrin (PE)-conjugated CD86, Allophycocyanin (APC)-conjugated CD80 antibodies for flow cytometry experiments were procured from BD Biosciences, United States. Anti-histone H3 polyclonal antibody (catalog no. PA516183) was procured from Invitrogen. Complement reagent serum (Sigma, catalog no. S1-100 ml), PLG (catalog no. SRP6518), goat anti-FH (catalog no. SAB2500260), rabbit anti-C4BP (catalog no. HPA000968), mouse anti-PLG (Sigma, catalog no. SAB1406263), and plasmin substrate, UPA (Sigma, catalog no. SRP6273).

### Cloning, Expression, and Purification of Recombinant Proteins

Low-passage virulent *Leptospira interrogans* serovar Pomona was cultured at 28°C in Ellinghausen–McCullough–Johnson–Harris (EMJH) medium (BD Difco™, United States) supplemented with 10% bovine serum albumin (BSA). Genomic DNA was isolated using kit. Genes coding for surface-exposed lipoproteins, *viz*., LenB, LenD, LenE, and Lsa30, were PCR-amplified using specific primer and successfully cloned into His-tagged Pet28a Small Ubiquitin-like Modifier (SUMO) vector. Genes coding for Lipl21 and Loa22 were cloned in pDest vector through Gateway technology following the manufacturer’s instructions. The sequence of the cloned gene was verified with T7 primers. The plasmid constructs (Pet28a SUMO-LenB, Pet28a SUMO-LenD, Pet28a SUMO-LenE, Pet28a SUMO-Lsa30, pDest-LipL21, and pDest-LipLoa22) were transformed into BL21 DE3, and the resulting transformants were grown at 37°C overnight as primary culture followed by secondary culture in 1 L of Luria Broth (LB) broth containing ampicillin (50 μg/ml) or kanamycin (50 μg/ml), and the expression of the protein was induced with 1 mM isopropyl β-D-1-thiogalactoside. The cells were harvested by centrifugation at 3,000 × g, and the cell pellet was resuspended in 100 mM Tris Cl and 150 mM Nacl (pH 8.0) and then sonicated at constant pulses. The lysate was centrifuged to remove cell debris, and the supernatant was subjected to affinity chromatography using Ni-Nitrilotriacetic acid (NTA) beads (GE Healthcare). The recombinant fusion proteins were eluted in 300 mM Imidazole in Phosphate buffered saline (PBS) (pH 7.4), whereas the protein was bound to the column by incubating at 4°C for 12–16 h. The proteins were eluted and checked for size and purity by Sodium dodecyl-sulfate polyacrylamide gel electrophoresis (SDS-PAGE). The concentration of purified protein was estimated using the Bradford reagent (Sigma B6916). The recombinant variable region of *Leptospira* immunoglobulin like protein A (LAV) was purified as described previously (Kumar et al., 2021).

### Cell Stimulation Assays

RAW264.7 cells (1 × 10^5^ cells per well) were cultured in a complete DMEM medium, and THP-1 cells (1 × 10^5^ cells per well) were cultured in a complete RPMI 1640 medium and differentiated to macrophage in the presence of 100-nm phorbol 12-myristate 12-acetate (PMA) for 3 days at 37°C and 5% CO_2_. After that, PMA was removed, and a fresh medium was added. BoMac cells (1 × 10^5^ cells per well) were cultured in a complete RPMI 1640 medium. Cells were stimulated with varying concentration (1, 2.5, and 5 μg/ml) of each protein rLenB, rLenD, rLenE, rLsa30, rLipl21, and rLoa22 or rSUMO for 24 h at 37°C in presence of 5% CO_2_. Cells stimulated with N-Palmitoyl-S-[2,3-bis(palmitoyloxy)-(2RS)-propyl]-[R]-cysteinyl-[S]-seryl-[S]-lysyl-[S]-lysyl-[S]-lysyl-[S]-lysine (PAM3CSK4) (20 ng/ml) and Lipopolysaccharide (LPS) (500 ng/ml) were used as positive controls, whereas those stimulated with media alone were considered negative control. The proteins were pre-treated with Polymyxin B (PMB; 10 μg/ml protein) at 37°C for 1 h before each assay to rule out endotoxin activity. To confirm that the observed effect was protein specific, samples were treated with Proteinase K (PK) (5 μg/mg protein) at 65°C for 1 h followed by inactivation of PK enzyme at 95°C for 5 min. The thermal stability of the protein or its degradation after PMB and PK treatment, respectively, was confirmed by SDS-PAGE.

### Isolation of Bone Marrow-Derived Macrophages

Bone marrow–derived macrophages (BMDMs) were generated following the published procedure (Zamboni and Rabinovitch, 2003). Bone marrow cells obtained from 6- to 8-week-old C57BL/6 mice were resuspended in 10 ml of bone marrow differentiation media (complete DMEM supplemented with 20% L929 cell–conditioned medium as a source of granulocyte/macrophage colony-stimulating factor). Cells were seeded in 100-mm dishes and incubated at 37°C in 5% CO_2_. Three days after seeding the cells, an extra 10 ml of fresh bone marrow differentiation media were added per plate and incubated for an additional 4 days. To harvest BMDM, the supernatant was removed and the attached cells were washed twice with complete DMEM. The macrophages were detached by gently pipetting the complete DMEM across the dish. The cells were centrifuged at 450 × g for 10 min and resuspended in 10 ml of complete BMDM. The cells were counted, seeded, and cultivated in tissue culture plates for 16 h and then used for specific assays.

### Cytokine ELISA

The culture supernatants from stimulated RAW264.7 and THP-1 cells were collected after 24 h, and cytokines (IL-6 and TNF-α) were measured using sandwich ELISA kits (R&D Systems) following the manufacturer’s protocol.

### RT-PCR

After treatment, RAW264.7 or BoMac cells were recovered in 500 μl of TRIzol (Invitrogen, Carlsbad, CA, United States), and equal volumes of chloroform were added; samples were centrifuged at 12,000 rpm for 15 min at 4°C. The aqueous phase was then passed through RNAeasy mini columns (MN), and RNA was purified following the manufacturer’s protocol. The RNA quantity was assessed by UV spectroscopy and purity by 260/280 ratio. First-strand cDNA was synthesized using the Takara cDNA synthesis kit (catalog no. 6110A) following the manufacturer’s instructions. RT-PCR was performed in 96-well microtiter plates in a 10-μl reaction volume containing 50 ng of cDNA, 10 μM each primer (Table 1), and SYBR green (Bio-Rad). Samples were run in triplicate, and data were analyzed with Sequence Detection System (Bio-Rad CFX-96). The experimental data were presented as fold changes of different cytokines and chemokines gene expression. RNA levels of the analyzed genes were normalized to the amount of Glyceraldehyde-3-Phosphate Dehydrogenase (GAPDH) present in each sample.

**TABLE 1:** Primers used for RT-PCR and gene amplification.

S. no	Gene	Primer sequence
1	GAPDH	F-GCCTGGAGAAACCTGCC R-ATACCAGGAAATGAGCTTGACA
2	iNOS	F-CAGCCCAACAATACAAGATGACCC R-CAGTTCCGAGCGTCAAAGACCTGC
3	CXCL10	F-CATGGTCCTGAGACAAAAGT R-TGATGACACAAGTTCTTCCA
4	IL-1b	F-GCCTTGGGCCTCAAAGGAAAGAATC R-GGAAGACACAGATTCCATGGTGAAG
5	CXCR3	F-GCCGGAGCACCAGCCAAGCCAT R-AGGTGGAGCAGGAAGGTGTC
6	CCR-4	F-ATCCTGAAGGACTTCAAGCTCCA R-AGGTCTGTGCAAGATCGTTTCATGG
7	CCL3	F-ACTGCCTGCTGCTTCTCCTACA R-AGGAAAATGACACCTGGCTGG
8	CXCR-4	F-GAAGTGGGGTCTGGAGACTATG R-AGGGGAGTGTGATGACAAAGAG
9	CCR-3	F-CAACTTGGCAATTTCTGACCTG R-GCAAACACAGCATGGACGATAG
10	COX-2	F-TCTGGAACATTGTGAACAACATC R-AAGCTCCTTATTTCCCTTCACAC
11	CCL-2	F-ACGTGTTGGCTCAGCCAGA R-ACTACAGCTTCCTTTGGGACACC
12	LenB	F-CGTGGATCCGGTACAAGAGAAGCGGTT R-TTCTCGAGTTACTGTTCTACACAGAGTAGATTC
13	LenD	F-ATTCGGATCCAAATCGAAATTTAAATTCGTTGC R-TTCTCGAGTTATTGTTCTACACAAACGACT
14	LenE	F-ACTGGATCCTACAATCAAACCGCTCTAAAT R-ATTCTCGAGTTATTGTTCAACGCATAGAAT
15	Lsa30	F-ATACTGGATCCATCCGAAACACAGTAATCC R-ATTCTCGAGTTAAAATAAATTACAACCAGTCTG
16	Lipl21	F-GGGGACAAGTTTGTACAAAAAAGCAG GCTTCATGATCAATAGACTTATAGCTCTATCTTTA R-GGGGACCACTTTGTACAAGAAAGCTGGGTTTTA TTGTTTGGAAACCTCTTGA
17	Loa22	F-GGGGACAAGTTTGTACAAAAAAGCAG GCTTCATCGTCAAAAAGATTTTGAATCTGAT R-GGGGACCACTTTGTACAAGAAAGCTGGGTTTTA TTGTTGT GGTGCGG
18	IL-6	F-TGCTTGATCAGAACCACTGC R-GCGATCTTTTGCTTCAGGAT
19	TNF-α	F-CCCCCAGAGGGAAGAGTCC R-GGGCTACCGGCTTGTTACTTG

### Flow Cytometry Analysis

RAW264.7 (10^6^ cells per well) were stimulated with LPS (500 ng/ml) or PMB-treated rLenB, LenD, rLenE, rLsa30, rLipl21, or rLoa22 for 24 h at 37°C in presence of 5% CO_2_. Cells were harvested and washed with prechilled PBS and after blocking; they were then incubated with PerCP-CY5.5–conjugated anti-MHCII, APC-conjugated CD80, and PE-conjugated CD86 (BD Biosciences, United States) for 1 h on ice in the dark. The cells were fixed with 1% paraformaldehyde, and 50,000 total events per sample were acquired using a LSRFortessa. The data were analyzed using FlowJo software.

### Pull-Down Assay

Recombinant proteins rLenB, rLenD, rLenE, rLsa30, rLipl21, rLoa22, or rLAV (positive control) along with 10% heat-inactivated normal human serum (NHS), were incubated with 15 μl of Ni-NTA agarose beads (Takara) overnight at 4°C. Only beads incubated with 10% heat-inactivated NHS was used as negative control. Agarose beads were washed five times with PBS, and then, interacting proteins were eluted with PBS containing 250 mM imidazole. Each elutes boiled in reducing Laemmli buffer and subjected to SDS-PAGE. The proteins were transferred to nitrocellulose membrane. The blot was blocked with 5% skimmed milk, washed, and then incubated with anti-FH, anti-C4BP, anti-PLG antibody diluted in 3% skimmed milk overnight at 4°C. The blots were washed and then incubated with anti-goat/anti-rabbit/anti-mouse IgG–horseradish peroxidase (HRP) at room temperature for 2 h. The blots were washed and then developed with ECL substrate (Bio-Rad).

### ELISA Binding Assay

The binding of proteins to soluble FH, C4BP, and PLG was analyzed by ELISA. Microtiter plates were coated overnight at 4°C with rLenB, rLenD, rLenE, rLsa30, rLipl21, or rLoa22 (1 μg/ml each). BSA was used as a negative control. The wells were washed three times with PBS containing 0.05% Tween 20 (PBS-T), blocked with 300 μl of PBS/2% BSA for 2 h at 37°C, and incubated with different concentrations (0–100%) of NHS diluted in PBS for 90 min at 37°C. After washing, appropriately diluted rabbit anti-C4BP, goat anti-FH, or mouse anti-PLG antibodies were added, and plates were further incubated for 1 h at 37°C. After washing the plates (three times), 100 μl of PBS containing HRP-conjugated anti-rabbit/goat/mouse IgG was added to each well, and plates were incubated for 1 h at 37°C. After usual washing, TMB (100 μl per well) was added, and reactions were allowed to proceed for 15 min and then stopped by adding 50 μl of 2N H_2_SO_4_. The plate was read at 450 nm in a microplate reader.

### Plasmin Activity Assay

Microtiter plate wells were coated overnight with 2 μg per well of rLenB, rLenD, rLenE, rLsa30, rLipl21, or rLoa22 at 4°C overnight. The wells were washed with PBS-T and blocked for 2 h at 37°C with 10% non-fat milk. The blocking solution was discarded, and human PLG (2 μg per well) was added, followed by incubation for 90 min at 37°C. Wells were washed three times with PBS-T, and then, urokinase PLG activator (uPA, 3 U per well) was added together with plasmin-specific substrate. D-Val-Leu-Lys 4-nitroanilide dihydrochloride in a plate was incubated overnight at 37°C. The absorbance was measured at 405 nm.

### Nuclease Activity

To examine the DNase activity of rLenB, rLenD, rLenE, rLsa30, rLipl21, and rLoa22, DNA fragment was incubated with 5 μg of concentration of each protein (rLenB, rLenD, rLenE, rLsa30, rLipl21, or rLoa22) or DNase I (20 IU, positive control) in Dulbecco’s Phosphate-Buffered Saline (DPBS) with 5 mM MgCl_2_, CaCl_2_, or ZnCl_2_ in a PCR tube at 37°C for 2 h. The reaction mixture was subjected to EtBr Agarose gel electrophoresis (1%) and observed under the Gel doc. Cleavage activity was quantified by measuring the ethidium bromide signal in each lane and calculating the fraction of DNA digested relative to the untreated DNA using ImageJ software.

### Isolation of Neutrophils From Murine Blood

Neutrophils were isolated from mouse blood using standard procedure (Swamydas et al., 2015). Blood was collected from four to five C57BL/6 mice by ocular puncture in tubes containing Ethylenediamine tetraacetic acid (EDTA). Blood (1 ml) pooled from different mice was mixed with Ammonium-Chloride-Potassium (ACK) lysis buffer to lyse RBCs. The cells were washed with RPMI 1640 supplemented with 10% FBS, counted, and re-suspended in 1 ml of ice-cold sterile PBS. Cells were overlaid on 3 ml of Histopaque 1077/1119 mix in a 15-ml conical tube and then centrifuged for 30 min at 825 × g at 25°C without braking. Neutrophils at the interface were collected and washed twice with a complete RPMI 1640 medium, counted, and suspended in the medium for the specific assay. The viability was determined by Trypan blue exclusion assay. The purity of neutrophils was determined by Flow cytometry (FACS) using anti-Ly6G antibody.

### Neutrophil Extracellular Trap Assay

NETosis was measured by quantifying elastase released from degraded NET DNA using the Neutrophil Elastase Activity Assay kit (catalog no. 600610, CaymanChemicals, United States) following the manufacturer’s protocol. Briefly, mouse neutrophils (1 × 10^5^ cells per well) were seeded in 96-well plate and treated with 3 μl of PMA (50 ng/ml) followed by incubation for 3 h at 37°C/5% CO_2_. After NET induction, the medium was replaced with 5 μg of protein (rLenB, rLenD, rLenE, rLsa30, rLipl21, or rLoa22) in nuclease assay buffer (PBS with 5 mM MgCl_2_, CaCl_2_, or ZnCl_2_) and incubated for 4 h at 37°C/5% CO_2_. DNase I and BSA were used as positive and negative control, respectively. At the end of treatment, 10 μl of supernatant was mixed with 90 μl of diluted assay buffer, and, finally, 10 μl of substrate solution (Z-Ala-Ala-Ala-Ala) was added. The total mixture was incubated in a black OPTI plate for 1.5 h at 37°C. The plates were read in a multi-mode reader at an excitation wavelength of 485 and 525 nm. In another experiment, 2 × 10^5^ freshly isolated neutrophils in 300 μl of medium were added to the imaging dish and kept at 37°C in the presence of 5% CO_2_ overnight. Cells were treated with 3 μl of PMA (50 ng/ml) and incubated for 3 h at 37°C/5% CO_2_. Cells were then washed thrice with DPBS and then incubated with 20 μg of rLenB, rLenD, rLenE, rLsa30, rLipl21, or rLoa22 or 20 IU of DNase I in PBS containing 5 mM cocktail of MgCl_2_, CaCl_2_, and ZnCl_2_ for 2 h at 37°C/5% CO_2_. Cells were washed with DPBS, fixed with 4% Paraformaldehyde (PFA) (15 min at room temperature) and then stained with anti-histone H3 antibody (1:250) overnight at 4°C. Cells were washed thoroughly with DPBS, mounted with VECTASHIELD containing 4′,6-diamidino-2-phenylindole (DAPI) mounting medium and observed using a 63 × oil objective on a confocal microscope (Leica SP8, Wetzlar, Germany).

### Statistical Analysis

For all the experiments, wherever required, GraphPad Prism 7.0 (GraphPad Software, Inc.) and one-way ANOVA using the Dunnett multiple comparisons were executed to analyze the results. The data were represented as the mean of triplicates ± SEM. *p* < 0.05 was considered significant.

## Results

### Cloning, Expression, and Purification of Recombinant Proteins

**FIGURE 1:**
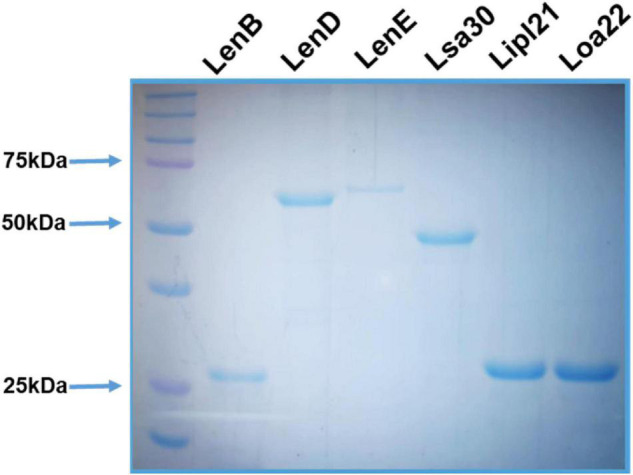
Purification of recombinant surface-exposed lipoproteins: The recombinant proteins were purified as described in section “Materials and Methods”. SDS-PAGE profile shows the pure LenB (27 kDa), LenD (62 kDa), LenE (65 kDa), Lsa30 (45 kDa), Lipl21 (21 kDa), and Loa22 (22 kDa).

The surface-exposed lipoproteins involved in pathogenesis, *viz*., Len proteins (LenB, LenD, and LenE), Lsa30, Loa22, and LipL21 were selected on the basis of previous studies as compiled in a review article by Raja and Natarajaseenivasan (2015). The genes coding for LenB, LenD, LenE, Lsa30, Lipl21, and Loa22 were PCR-amplified, cloned, expressed, and purified from soluble fractions. The SDS-PAGE profile in Figure 1 shows that proteins were pure with an expected molecular weight of LenB (27 kDa), LenD (62 kDa), LenE (65 kDa), Lsa30 (45 kDa), Lipl21 (21 kDa), and Loa22 (22 kDa).

### Screening of Recombinant Proteins for Their Innate Immune Activity

**FIGURE 2:**
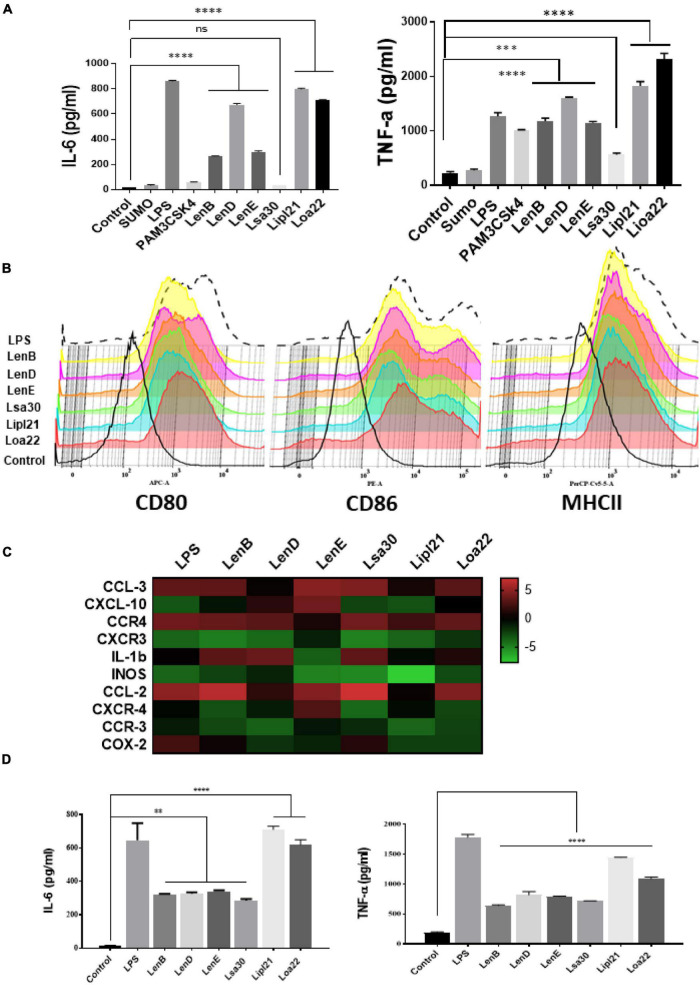
Analysis of activation of mouse macrophages after stimulation with surface proteins. **(A)** Screening of pro-inflammatory response of surface proteins in RAW264.7 cells by ELISA. RAW264.7 cell lines were stimulated with LPS-treated (500 ng/ml), PAM3CSK4-treated (20 ng/ml), or PMB-treated (1μg/ml) rLenB, rLenD, rLenE, rLsa30, rLipl21, rLoa22, or rSUMO for 24 h at 37°C/5% CO_2_, and supernatant was collected to measure levels of IL-6 and TNF-α using sandwich ELISA kit. **(B)** Expression of surface markers in RAW cells after stimulation with surface proteins. RAW264.7 cell lines were stimulated with LPS-treated (500 ng/ml) or PMB-treated (1μg/ml) rLenB, rLenD, rLenE, rLsa30, rLipl21, or rLoa22 for 24 h at 37°C/5% CO_2_. Cells were stained with fluorochrome-conjugated antibodies against CD80, CD86, and MHCII and then analyzed by flow cytometry as described in section “Materials and Methods”. **(C)** Analysis of expression of immune response-related genes in mouse macrophages stimulated with surface proteins. RAW264.7 cells were treated with LPS (500 ng/ml) or PMB (1 μg/ml) of each protein (rLenB, rLenD, rLenE, rLsa30, rLipl21, or rLoa22) for 24 h at 37°C/5% CO_2_. Cells were recovered, RNA was isolated and converted to cDNA, and gene expression was analyzed by RT-PCR using specific primers as described in section “Materials and Methods”. The data were presented as fold changes between stimulated cells vs. control and normalized to GAPDH. **(D)** Analysis of activation of mouse bone marrow–derived macrophages (BMDM) after stimulation with surface proteins. BMDMs were isolated as described in section “Materials and Methods”. Cells were stimulated with LPS-treated (500 ng/ml) or PMB-treated (1 μg/ml) rLenB, rLenD, rLenE, rLsa30, rLipl21, or rLoa22 for 24 h at 37°C/5% CO_2_, and supernatant was collected to measure levels of IL-6 and TNF-α using sandwich ELISA kit. Data are representative of three different experiments. Significant differences were calculated using the one-way ANOVA (****, ***, **, and ns indicates *P* < 0.0001, *P* < 0.001, *P* < 0.001, and non-significant, respectively).

**FIGURE 3:**
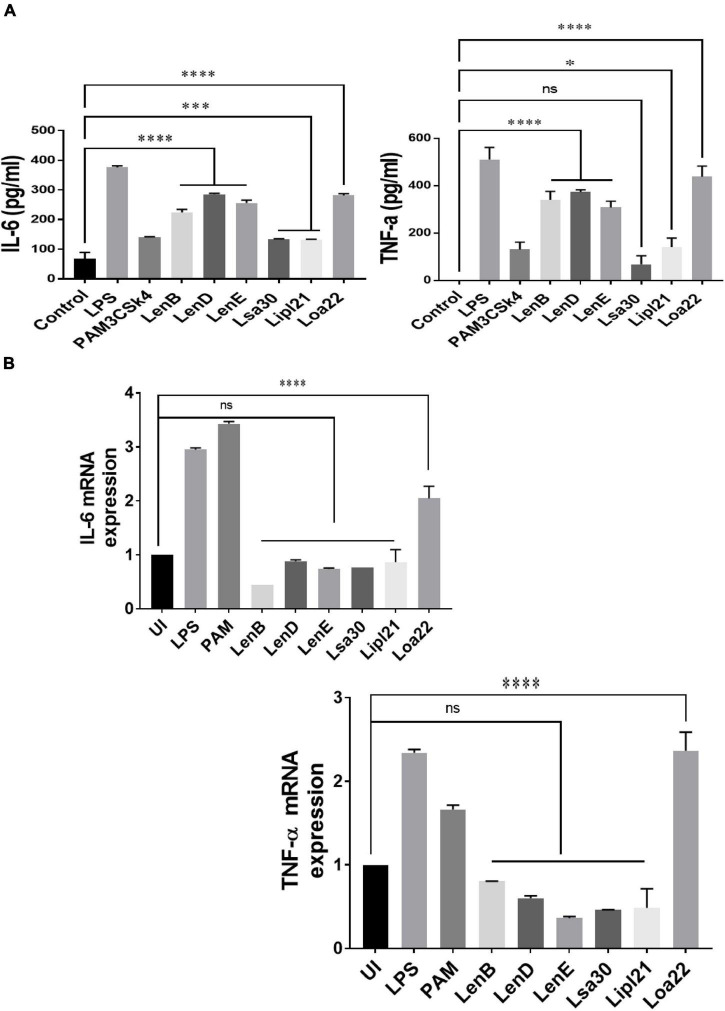
Proinflmmatory effects of surface proteins on human and bovine macrophages. **(A)** Screening of pro-inflammatory response of surface proteins in THP-1 cells by ELISA. PMA-differentiated THP-1 cells were stimulated with LPS (500 ng/ml) or PAM3CSK4 (20 ng/ml) or PMB-treated (1 μg/ml) rLenB, rLenD, rLenE, rLsa30, rLipl21, or rLoa22 for 24 h at 37°C/5% CO_2_, and supernatant was collected to measure levels of TNF-α and IL-6 using sandwich ELISA kit. **(B)** Screening of pro-inflammatory response of surface proteins in Bomac cells by RT-PCR. BoMac cells were cultured and stimulated with proteins as described above. Cells were harvested, RNA was isolated, and cDNA was converted. RT-PCR was performed using specific primers of bovine IL-6 and TNF-a as described in section “Materials and Methods”. Data are representative of three different experiments. Significant differences were calculated using the one-way ANOVA (****, ***, *, and ns indicates *P* < 0.0001, *P* < 0.001, *P* < 0.05, and non-significant, respectively).

To evaluate the innate immune activity of rLenB, rLenD, rLenE, rLsa30, rLipl21, and rLoa22, we tested their ability to activate mouse macrophages. We stimulated mouse macrophages (RAW264.7) with varying doses (1, 2.5, and 5 μg/ml) of the proteins (rLenB, rLenD, rLenE, rLsa30, rLipl21, or rLoa22). Our result shows that, of various proteins tested, Loa22 induced a strong pro-inflammatory response and Lsa30 was least stimulatory as evident by induction of IL-6 and TNF-α (Figure 2A). Further, these proteins induced concentration-dependent pro-inflammatory response (Supplementary Figure 1). To confirm whether stimulation with these proteins causes macrophages activation and maturation, we analyzed the expression of costimulatory molecules (CD80 and CD86) and maturation marker (MHCII) in RAW264.7 cells. All proteins induced the expression of CD80, CD86, and MHCII; however, maximum expression was induced by Loa22 followed by the Len family of proteins (Figure 2B and Supplementary Figure 2). To rule out that the observed pro-inflammatory effect was protein specific and not due to contaminating LPS, each protein was passed through PMB-agarose and was also pre-incubated with PMB in cell stimulation assays. LPS (500 or 1 μg/ml) pre-incubated with PMB was used as a control to check the potency of PMB. The estimated concentration of LPS in final protein preparation varied from (0.10–0.15 ng/ml) (data not shown). Our result shows that PK plus heating abolished cytokine production and inhibited expression of surface markers. Further, PMB inhibited the LPS-induced cytokine production and expression of surface markers but did not attenuate the levels of cytokines or inhibited expression of markers induced by proteins (Supplementary Figures 3, 4). These results indicate that the stimulatory effects observed were specific to protein and not due to contamination with LPS. Because these proteins were sumo fusion proteins, we used sumo protein as control, which did not show any significant effect. To check whether, apart from IL-6 and TNF-α, these proteins can induce expression of other cytokines, chemokines, or their receptors, we did gene expression analysis of select genes by RT-PCR. Our result shows that all the proteins induced expression of several chemokines (CCL2, CCL3, and CXCL10), their receptors (CCR3, CCR4, CXCR3, and CXCR4), and other proinflammatory cytokines like IL-1β (Figure 2C). To check whether these proteins can activate primary innate cells, we stimulated BMDMs and analyzed the proinflammatory cytokines. Our result shows that, similar to RAW264.7 cells, these proteins stimulated BMDMs to produce significant levels of IL-6 and TNF-α (Figure 2D). To test whether these surface proteins can induce activation of macrophages from natural hosts (human and bovine), we stimulated THP-1 and BoMAC cells and analyzed proinflammatory cytokines. Our result shows that, of all proteins tested, Loa22 induced significant activation of both human and bovine cells. LipL21, which induced a strong response in mouse macrophages, was able to induce low or insignificant level of activation indicating that the response was attenuated in both human and bovine macrophages (Figure 3). Together, these results indicate that these surface-exposed lipoproteins can induce innate immune activation.

### Screening of Surface Proteins for Their Ability to Bind to Complement Regulators (**Factor H** and C4BP) and Host Proteases (Plasminogen)

**FIGURE 4:**
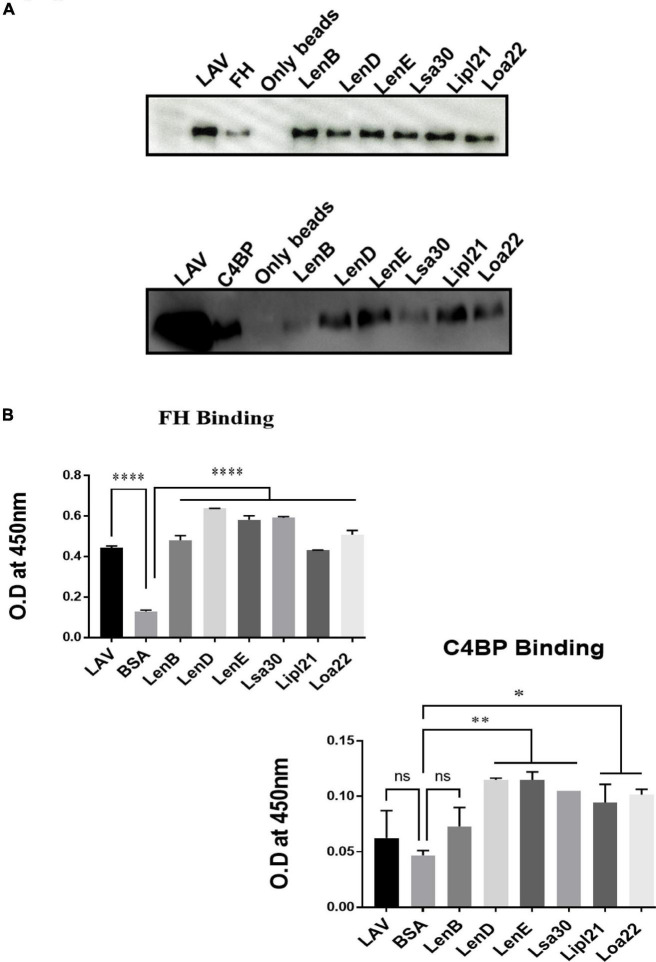
Evaluation of binding of surface proteins with complement regulators (FH and C4BP). **(A)** Binding of surface proteins with FH and C4BP as analyzed by pull-down assay. Bead bound proteins (rLenB, rLenD, rLenE, rLsa30, rLipl21, rLoa22, or rLAV; 1 μg each) or only beads were incubated with 10% HI-NHS, and protein–protein interaction was detected using anti-FH or anti-C4BP antibodies by Western blot as described in section “Materials and Methods”. **(B)** Binding of surface proteins as analyzed by ELISA. Microtiter plates were coated with proteins (1 μg/ml) or BSA (negative control) followed by addition of 10% HI-NHS, and binding was detected with specific antibodies against FH and C4BP as described in section “Materials and Methods”. All data are representative of three different experiments. Significant differences were calculated using the one-way ANOVA (****, **, *, and ns indicates *P* < 0.0001, *P* < 0.01, *P* < 0.05, and non-significant, respectively).

**FIGURE 5:**
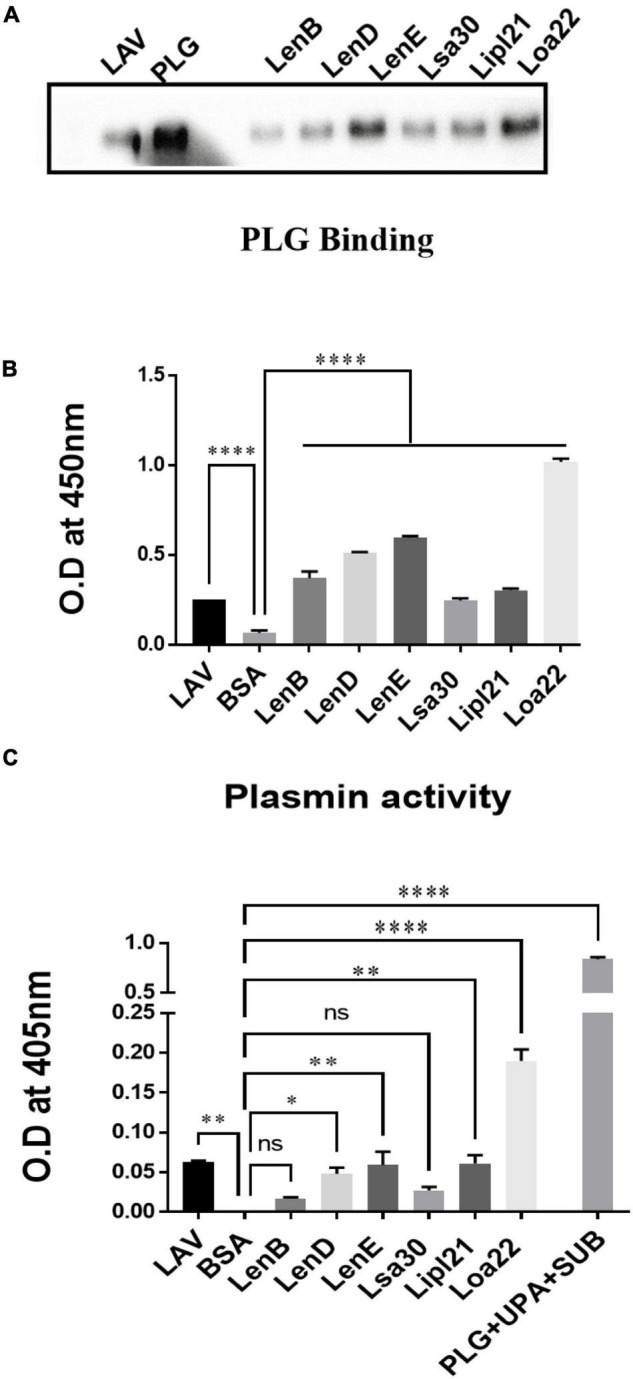
Evaluation of binding of surface proteins with host protease Plasminogen (PLG). **(A)** Binding of surface proteins with Plasminogen (PLG) as analyzed by pull-down assay. Bead bound proteins (rLenB, rLenD, rLenE, rLsa30, rLipl21, rLoa22, or rLAV; 1 μg each) or only beads were incubated with 10% HI-NHS (as a source of PLG), and protein–protein interaction was detected by western blot using anti-PLG antibody as described in section “Materials and Methods.”. **(B)** Binding of surface proteins analyzed by ELISA. Microtiter plates were coated with proteins (1 μg/ml) or BSA, and binding was detected with anti-PLG antibody as described in section “Materials and Methods”. **(C)** Plasmin activity. Surface proteins (2 μg per well) or BSA or LAV (2 μg per well) were immobilized on microtiter plates followed by the addition of PLG, uPA, and specific plasmin substrate. The plate was incubated overnight, and absorbance was read at 405 nm as described in section “Materials and Methods”. All data are representative of three different experiments. Significant differences were calculated using the one-way ANOVA (****, **, *, and ns indicates *P* < 0.0001, *P* < 0.01, *P* < 0.05, and non-significant, respectively).

Previous studies have shown that several surface proteins, including LenB, LenD, LenE, Lsa30, and Loa22 of *Leptospira*, bind to complement regulators like FH or C4BP. However, binding of these proteins was tested with any complement regular either FH or C4BP, and binding of the second most abundant protein, LipL21, has not been tested yet. In this study, we screened these proteins (LenB, LenD, LenE, Lsa30, LipL21, and Loa22) for binding with both FH and C4BP. We also assessed their ability to bind host proteases (PLG) and mediate subsequent plasmin activity. Our pull-down assay result shows that all the proteins, including LipL21, were able to bind to FH and C4BP (Figure 4A). The variable region of LigA (LAV), which was used as a positive control, showed binding with both FH and C4BP, whereas BSA, which was used as a negative control, did not show any significant level of binding (Figure 4A). This was further confirmed by ELISA, where the binding was enhanced with increasing concentration (0–100%) of NHS (Figure 4B and Supplementary Figure 5). Further, all proteins showed binding with PLG, which was enhanced with an increase in the concentration of NHS (Figure 5A and Supplementary Figure 5). Furthermore, the binding of proteins to PLG correlated to plasmin activity (Figure 5B). Among the proteins tested, Loa22 demonstrated the strongest binding affinity to PLG correlating to a generation of significantly higher levels of plasmin (Figure 5B).

### Screening of Surface Proteins for Nuclease Activity to Evaluate Their Potential Role in Evasion From Neutrophil Extracellular Traps

**FIGURE 6:**
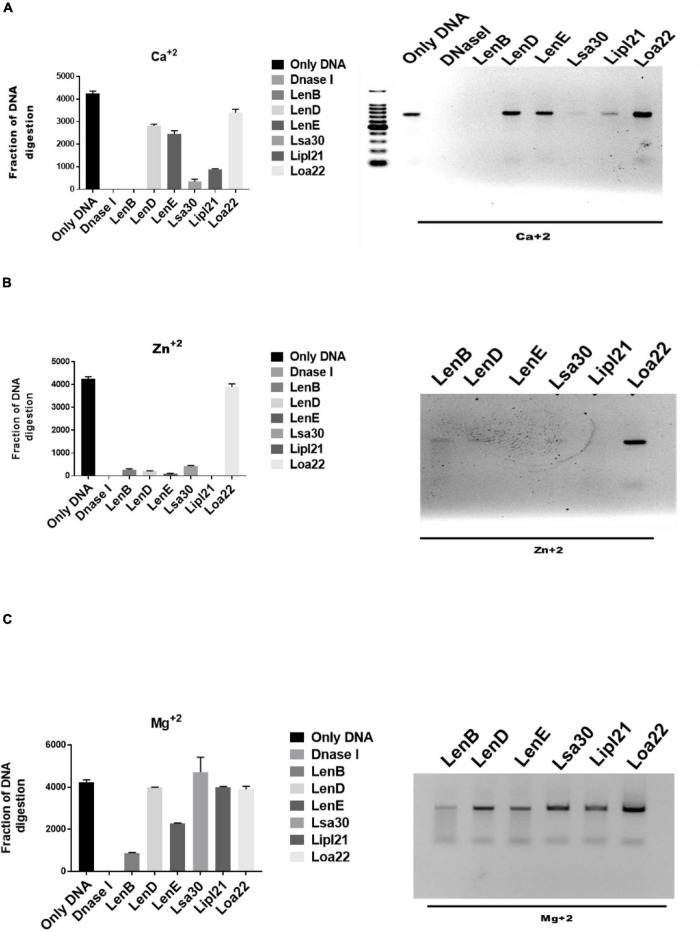
Evaluation of *in vitro* nuclease activity of surface-exposed lipoproteins. Seven hundred–base pair DNA fragment (200 ng) was incubated with 5 μg of surface proteins (rLenB, rLenD, rLenE, rLsa30, rLipl21, or rLoa22) or DNase I in DPBS with 5 mM MgCl_2_, CaCl_2_, or ZnCl_2_ at 37°C for 3 h followed by visualization using the Agarose gel electrophoresis. Screening of nuclease activity of surface proteins in presence of **(A)** calcium chloride, **(B)** zinc chloride, and **(C)** magnesium chloride. All data are representative of three different experiments.

**FIGURE 7:**
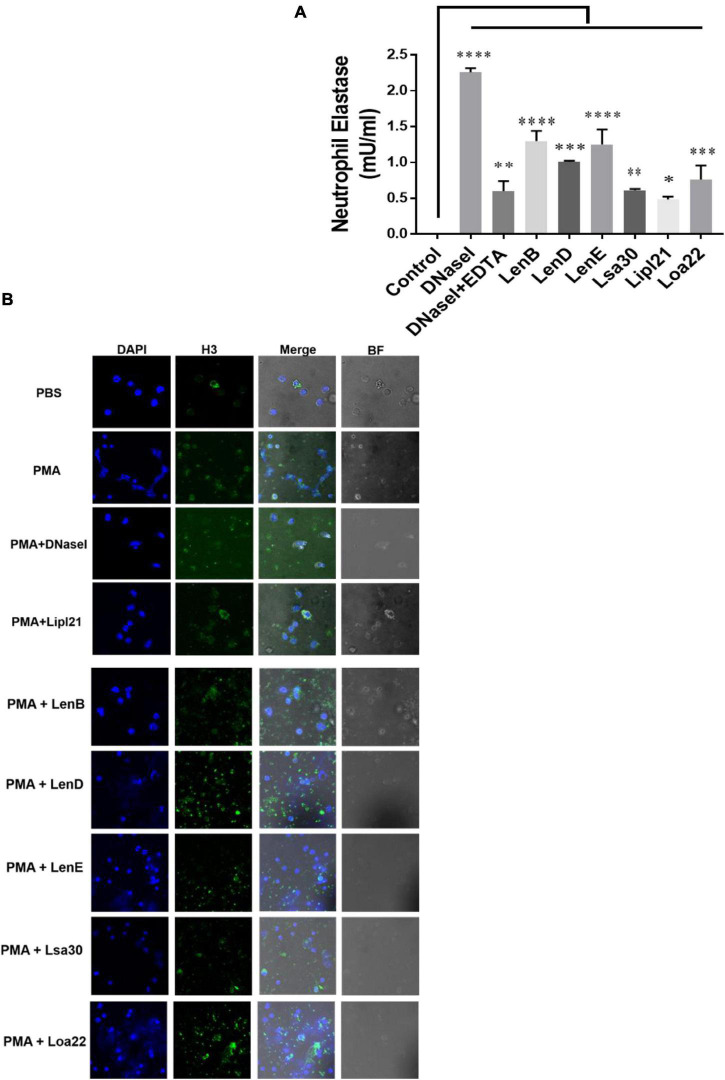
Evaluation of surface proteins ability to degrade neutrophil extracellular trap (NET). **(A)** Evaluation of NETosis by elastase activity. Purified neutrophils from mouse blood were stimulated with PMA to induce NET, followed by incubation with 5 μg of protein in nuclease assay buffer for 4 h at 37°C/5% CO_2_ as described in section “Materials and Methods”. Elastase activity was determined by using a neutrophil elastase activity kit following the manufacturer’s instruction. Data are representative of three different experiments. Significant differences were calculated using the one-way ANOVA (****, ***, **, *, and ns indicates *P* < 0.0001, *P* < 0.001, *P* < 0.01, *P* < 0.05, and non-significant, respectively). **(B)** Evaluation of NETosis by Confocal microscopy. Neutrophils (2 × 10^5^) were treated with PMA (50 ng/ml) and incubated for 3 h at 37°C/5% CO_2_. After washing cells were treated with 20 μg of rLenB, rLenD, rLenE, rLsa30, rLipl21, or rLoa22 or 20 IU of DNase I in PBS containing 5 mM cocktail of MgCl_2_, CaCl_2_, and ZnCl_2_ for 2 h at 37°C/5% CO_2_. Cells were washed and then stained with anti-Histone H3 antibody (1:250) overnight at 4°C, mounted with VECTASHIELD (with DAPI) and observed using a 63 × oil objective on a confocal microscope as described in section “Materials and Methods”. BF, bright field.

Several bacteria express nucleases to escape from the NET. *Leptospira* is known to induce NET; however, whether they express nuclease to degrade NET is unknown. To identify such nucleases, we screened surface-exposed lipoproteins, LenB, LenD, LenE, Lsa30, LipL21, and Loa22, for their nuclease activity by incubating the DNA fragment with each protein (5 μg) along with different metal ions (divalent cations) like Ca2 +, Zn2 +, and Mg2 +. Cleavage activity was quantified by measuring the ethidium bromide signal in each lane and calculating the fraction of DNA digested relative to the untreated DNA. Our result shows that only LenB, Lsa30, and LipL21 exhibited significant nuclease activity in the presence of Ca2 +, whereas all the proteins, except Loa22, exhibited nuclease activity in the presence of Zn2 + (Figure 6). LenB and partially LenE exhibited nuclease activity in the presence of Mg2 +. LenD and LenE showed a strong activity in the presence of Zn2 + but weak activity in the presence of Ca2 + and Mg2 + (Figure 6). LenB showed nuclease activity in the presence of all metal ions, whereas Loa22 did not show any nuclease activity irrespective of metal ions used (Figure 6). To correlate the nuclease activity with *Leptospira* pathogenesis, we tested the ability of these proteins to degrade PMA induced NET *in vitro*. Our result shows that all the proteins, including Loa22, were able to degrade NET as evidenced by the increase in levels of elastase due to its release after treatment with protein (Figure 7A). The elastase levels were enhanced due to treatment with DNase, which was significantly reduced when DNAse was pre-treated with EDTA (a chelating agent), indicating the inhibition of nuclease activity (Figure 7A). NET degration was also confirmed by confocal microscopy. Our confocal microscopy result shows that LipL21, LenB, LenD, LenE, and Lsa30 could degrade the PMA induced NET; however, Loa22 was able to degrade to a lesser extent. These results confirm that these surface proteins have nuclease activity and a possible role in degrading NETs *in vivo* (Figure 7B).

## Discussion

Surface-exposed lipoproteins of *Leptospira* play a significant role in facilitating its survival in different environmental conditions and hosts for prolonged periods (Haake, 2000). Several surface-exposed lipoproteins of *Leptospira* have either not been characterized or are of unknown function (Raja and Natarajaseenivasan, 2015). The bacteria exploit several of these proteins to circumvent host-mediated killing at various stages of infection. These proteins may act as an adhesin and bind to host extracellular matrix molecules like fibronectin, laminin, and collagen, thereby helping bacteria to attach to the host cells to initiate infection (Christodoulides et al., 2017). They may activate the innate response *via* signaling through TLRs like TLR2 or TLR4, inducing a pro-inflammatory response, which may favor bacterial pathogenesis (Yang et al., 2006, Yang, 2007; Wang et al., 2012; Faisal et al., 2016). Alternatively, to evade this innate recognition *Leptospira* like another bacterial pathogen, may downregulate the expression or cause antigenic variations in their surface molecules including proteins upon infection in host (Cox et al., 1992; Zhang and Norris, 1998; Bunikis and Barbour, 1999; Xue et al., 2010). Further, the bacteria may utilize these proteins to evade from complement attack by binding to complement regulators like FH and C4BP or acquiring host proteases like PLG, which cleaves complement component (Verma et al., 2006; Stevenson et al., 2007; Castiblanco-Valencia et al., 2012; Souza et al., 2012; Vieira et al., 2012, 2014; Fraga et al., 2016). *Leptospira* like other bacterial pathogens may exploit these proteins to modulate function of phagocytes like neutrophils by inducing apoptosis, inhibiting phagocytosis and production of ROS or degrading NETs (Kinkead et al., 2018; Kobayashi et al., 2018; Vieira et al., 2018; Ledo et al., 2020). Several surface proteins of *Leptospira* have shown binding to FH, C4BP, and PLG and mediate co-factor activity, thereby indicating their possible role in evasion from complement-mediated killing (Verma et al., 2006; Stevenson et al., 2007; Castiblanco-Valencia et al., 2012; Souza et al., 2012; Wolff et al., 2013; Breda et al., 2015; da Silva et al., 2015; Fraga et al., 2016; Lin et al., 2016; Siqueira et al., 2016a,b; Amamura et al., 2017; Salazar et al., 2017; Kochi et al., 2019; Barbosa and Isaac, 2020). Until now, only LipL21 is known to modulate neutrophil function by inhibiting MPO activity (Vieira et al., 2018). As reported in other bacterial species, surface proteins that act as nucleases and able to degrade NET have not been reported in *Leptospira* (Kiedrowski et al., 2014; Morita et al., 2014; Dang et al., 2016; Binnenkade et al., 2018). Despite identification and characterization of several surface proteins of *Leptospira* involved in innate activation and/or complement evasion, not much effort is made toward the identification of immunomodulatory surface proteins, which are simultaneously involved in both immune activation and evasion. Recently, our group has demonstrated the immunomodulatory role of *Leptospira* immunoglobulin-like protein A (LigA) and characterized the domain involved in activation and evasion from host innate immune response (Kumar et al., 2021). Considering the functional redundancy in Leptospira surface proteins, in the present study, we aimed to identify and characterize the surface-exposed lipoproteins having this immunomodulatory or dual role that may help in better understanding their critical role in host–pathogen interaction. On the basis of the review of previously published studies, we selected few proteins from *Leptospira* endostatin like protein family (LenB, LenD, and LenE), *Leptospira* surface adhesin (Lsa30), 21kd Lipoprotein (LipL21), and OmpA-like protein (Loa22) that are known be involved in pathogenesis (Raja and Natarajaseenivasan, 2015). We then deciphered their role in immunomodulation in terms of their ability to activate innate immune cells or evade from complement system or phagocytes.

Our result shows that, of various proteins tested, Loa22 induced strong activation of macrophages of all hosts (mouse, human, and bovine), whereas Lsa30 was least stimulatory in terms of cytokine production (Figure 2A and Supplementary Figure 1). This activation also correlated to enhanced expression of costimulatory molecules (CD80 and CD86) and maturation marker (MHCII) (Figure 2B). Further, our RT-PCR result indicates that these proteins modulated the expression of several innate response related genes (cytokines, chemokines, and their receptors) involved in the activation and maturation of macrophages (Figure 2C). These results correlate to previous studies including a recent one by Vernel-Pauillac and Merien (2006); Domingos et al. (2017), and Shetty et al. (2021), showing that cytokines and chemokines play major role in early recruitment of innate cells like macrophages and dendritic cells (DCs) and may contribute to pathogenesis or controlling the infection. Previous studies have shown pro-inflammatory effects of surface proteins of *Leptospira*, mainly on mouse macrophages. However, the ability of these proteins to activate macrophages of susceptible hosts (human and bovine) has not been tested yet (Wang et al., 2012; Faisal et al., 2016; Hsu et al., 2021). In our study, we compared the innate activity of these proteins in macrophages of susceptible hosts (human and bovine), and our result shows that the stimulatory effects of these proteins varied in different host macrophages. Further, the response against LipL21 in Human THP-1 and BoMac cells was attenuated, as revealed by the production of significantly lower levels of pro-inflammatory cytokines (Figure 3). One can argue that the pro-inflammatory effect of these recombinant proteins might be due to the low amount of contaminating LPS; however, the use of proper controls like PMB and PK in our assay has ruled out this possibility and gives us confidence that the observed stimulatory effect is attributed to an inherent property of these proteins (Supplementary Figures 3, 4). The activation of macrophages by these surface-exposed lipoproteins might be TLR dependent, possibly through TLR2 as they are lipoproteins. However, this needs to be confirmed by additional experiments. Previous studies have shown that several recombinant surface proteins from both *Leptospira*, and other bacterial pathogens have shown to activate macrophages *via* signaling through TLR2 or TLR4 (Byun et al., 2012a,b,c; Sjolinder et al., 2012; Wang et al., 2012; Choi et al., 2016; Faisal et al., 2016). Loa22 has been shown to induce pro-inflammatory response *via* signaling through TLR2 (Hsu et al., 2021), whereas LipL21, by its binding to peptidoglycan, has been shown to be involved in immune response evasion by escaping recognition from NLRs (Ratet et al., 2017). Ongoing work in our laboratory is focused on identifying the host receptor involved in recognizing these lipoproteins, leading to subsequent activation of innate immune cells like macrophages and DCs. Further, innate immune system is not restricted to macrophages and neutrophils; hence, it would be interesting to see the effect of these proteins on other innate cells like DCs, monocytes, NK cells, and other myeloid cells as recent study has demonstrated the early recruitment of these cells during *Leptospira* infection (Shetty et al., 2021).

*Leptospira* is known to evade complement attack *via* binding complement regulators, secretion of self-proteases, or acquiring host proteases to degrade complement components (Amamura et al., 2017). Several surface-exposed lipoproteins of *Leptospira* apart from binding to components of host extracellular matrix have also been reported to bind to complement regulators and host proteases like PLG (Siqueira et al., 2016a,b; Kochi et al., 2019). Previous studies have shown binding of LenA and LenB to FH and Lsa30 to C4BP and PLG (Stevenson et al., 2007; Souza et al., 2012). The binding of other members of Len family (LenC, LenD, LenE, and LenF), LipL21 and Loa22 with complement regulators, or PLG has not been evaluated yet. Our study confirms the previous reports and demonstrates the binding of these proteins to both FH and C4BP and host proteases PLG, thereby highlighting their possible role in evading complement-mediated killing *via* inhibiting both classical and alternate pathways (Figures 4, 5).

Phagocytes like neutrophils have evolved a novel mechanism of killing the bacteria in extracellular space by throwing their DNA, histones, and antimicrobial peptides referred to as NETs (Brinkmann et al., 2004). This mechanism of killing extracellular bacteria by trapping outside the cell is called NETosis and is independent of phagocytosis and degranulation (McDonald et al., 2012). However, pathogens have evolved strategies to suppress or degrade NETs by expressing surface proteins having nuclease activity (Kaplan and Radic, 2012). Upon encounter with neutrophils, *Leptospira* can induce NET; however, to evade NETosis, whether it expresses nucleases has not been reported yet (Scharrig et al., 2015). For the first time, our group reported the role of LigA in degrading NET by its nuclease activity (Kumar et al., 2021). Considering the functional redundancy in surface proteins of *Leptospira*, it is likely that other surface-exposed lipoproteins might also possess nuclease activity. Keeping this in view, we tested the nuclease activity of LenB, LenD, LenE, Lsa30, LipL21, and Loa22. Our result showed that all the proteins, except Loa22, exhibited nuclease activity *in vitro*, albeit with the requirement of different metal ions (Figure 6). Further, the nuclease activity of these proteins correlated to their ability in degrading NET *in vitro* and thus highlights their possible role in escaping the *Leptospira* from NETosis (Figure 7). Although Loa22 did not show any nuclease activity *in vitro*, it was able to degrade NET, as evident from elastase activity and confocal microscopy (Figure 7). We speculate that Loa22, apart from having exonuclease activity, might also possess endonuclease activity; however, this needs further investigation. LipL21 can modulate neutrophil function by inhibiting MPO; hence, it is likely that apart from nuclease activity, these proteins might have a role in modulating neutrophil function by inhibiting ROS, MPO, and phagocytosis or by inducing apoptosis. However, these aspects need to be tested.

In conclusion, our results demonstrate that surface-exposed lipoproteins of *Leptospira* tested in this study have a role in modulating the host innate response by inducing pro-inflammatory effect, binding to complement regulators and host proteases to evade from complement attack and degrading NET by virtue of their nuclease activity. Although our *in vitro* results provide clear insight into the immunomodulatory function of these proteins, technical hurdles in validating them *in vivo* are the major limitation in claiming such phenomenon. However, the identification and characterization of such immunomodulatory proteins are of significant importance in understanding the *Leptospira* pathogenesis. Further, deciphering the immunomodulatory or dual role (immune activation and evasion) of these proteins may also help to understand how the bacteria disseminate and colonize in various organs, which may provide important insight into clinical outcomes in multiple hosts.

## Data Availability Statement

The original contributions presented in the study are included in the article/Supplementary Material, further inquiries can be directed to the corresponding author/s.

## Ethics Statement

The animal study was reviewed and approved by the Institutional Animal Ethics Committee of NIAB.

## Author Contributions

SMF conceived the idea and designed the experiments. AK and VPV performed the experiments and edited the manuscript. AK, VPV, and SMF analyzed the data. AK and SMF wrote the initial draft. All authors approved the final version of the manuscript.

## Conflict of Interest

The authors declare that the research was conducted in the absence of any commercial or financial relationships that could be construed as a potential conflict of interest.

## Publisher’s Note

All claims expressed in this article are solely those of the authors and do not necessarily represent those of their affiliated organizations, or those of the publisher, the editors and the reviewers. Any product that may be evaluated in this article, or claim that may be made by its manufacturer, is not guaranteed or endorsed by the publisher.
