# Supplementary material for: Screening of Surface-Exposed Lipoproteins of Leptospira Involved in Modulation of Host Innate Immune Response
Source: Front Microbiol. 2022 Mar 24;13:761670. doi: 10.3389/fmicb.2022.761670 (PMC8988195; doi:10.3389/fmicb.2022.761670)
Supplement: Supplementary file 1 [file Presentation_1.pdf]

Supplementary Materials

Title: Screening of surface exposed lipoproteins of *Leptospira* involved in modulation of host innate immune response.

Authors: Ajay Kumar ,Vivek P. Varma, Syed M. Faisal*

*Correspondence to: faisal@niab.org.in or smfaisal77@gmail.com

The supplementary file contains-

Figure S1- Analysis of pro-inflammatory cytokines after stimulation of RAW264.7 and THP-1 cells with varying concentrations of recombinant proteins.

Figure S2- Bar graph showing expression of surface markers in RAW264.7 cells after stimulation with surface proteins.

Figure S3- Effect of Protease K (PK) and Polymixin B (PMB) on protein and LPS induced production of IL-6 by RAW264.7 cells.

Figure S4- Effect of Protease K (PK) and Polymixin B (PMB) on protein and LPS induced expression of surface molecules by RAW264.7 cells.

Figure S5- Evaluation of binding of surface proteins with FH, C4BP and PLG in varying concentration of NHS.

Sup. Fig. 1

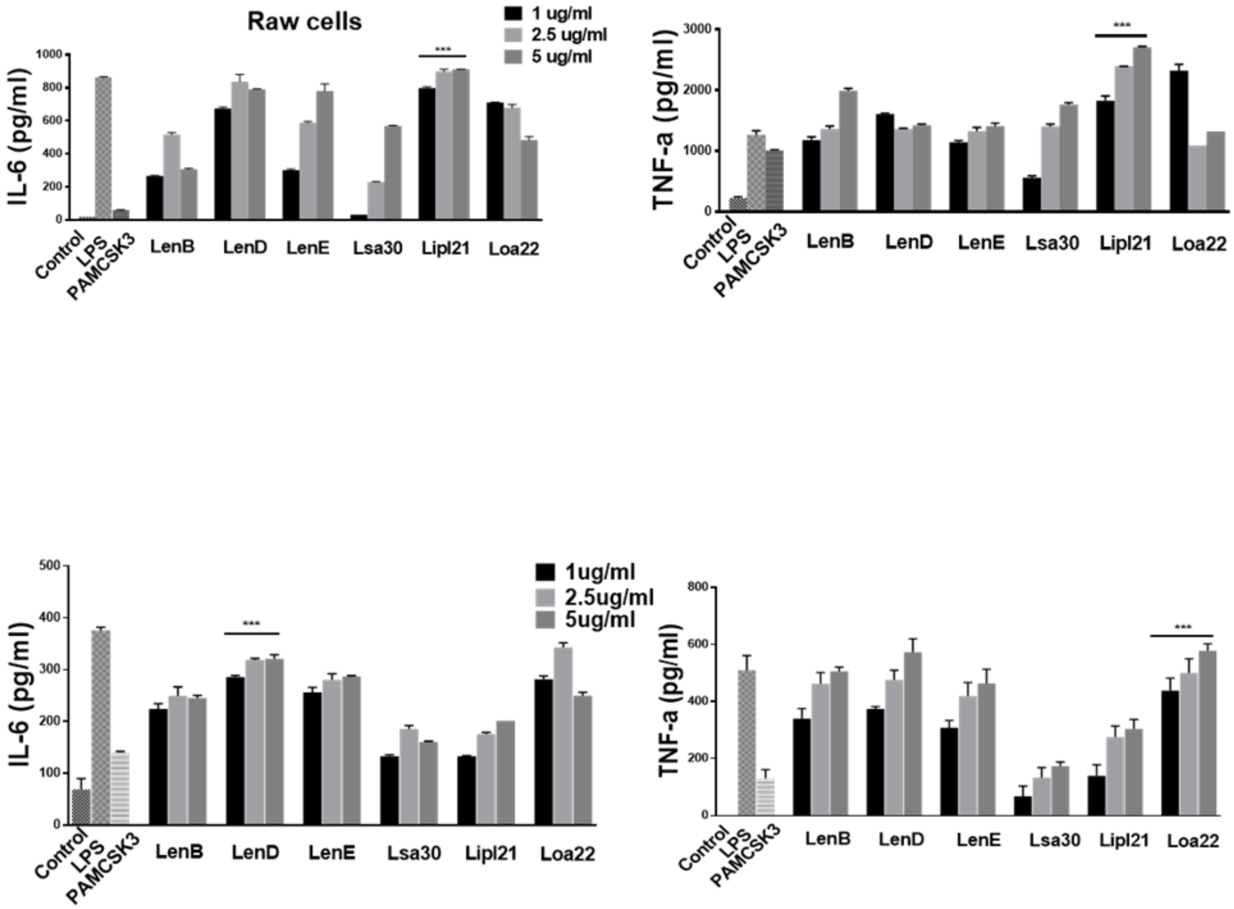

Sup. Fig. 1: Analysis of pro-inflammatory cytokines after stimulation of RAW264.7 and THP-1 cells with varying concentrations of recombinant proteins. (A) C*ytokine analysis in the culture supernatant of RAW cells stimulated with various concentrations of recombinant proteins*. The cells were stimulated with LPS (500ng/ml) or PMB treated varying concentrations (1, 2.5 and 5ug/ml) of rLenB or rLenD or rLenE or rLsa30 or rLipl21 or rLoa22 for 24h and supernatant was collected to measure levels of IL-6 and TNF-αusing sandwich ELISA kit. (B) C*ytokine analysis in the culture supernatant of THP-1 cells stimulated with various concentrations of recombinant proteins*. The cells were stimulated, and cytokines were analyzed as described above. Data are representative of three different experiments. Significant differences were calculated using the two-way ANOVA (*** indicates; $P < 0.001$).

Sup. Fig. 2

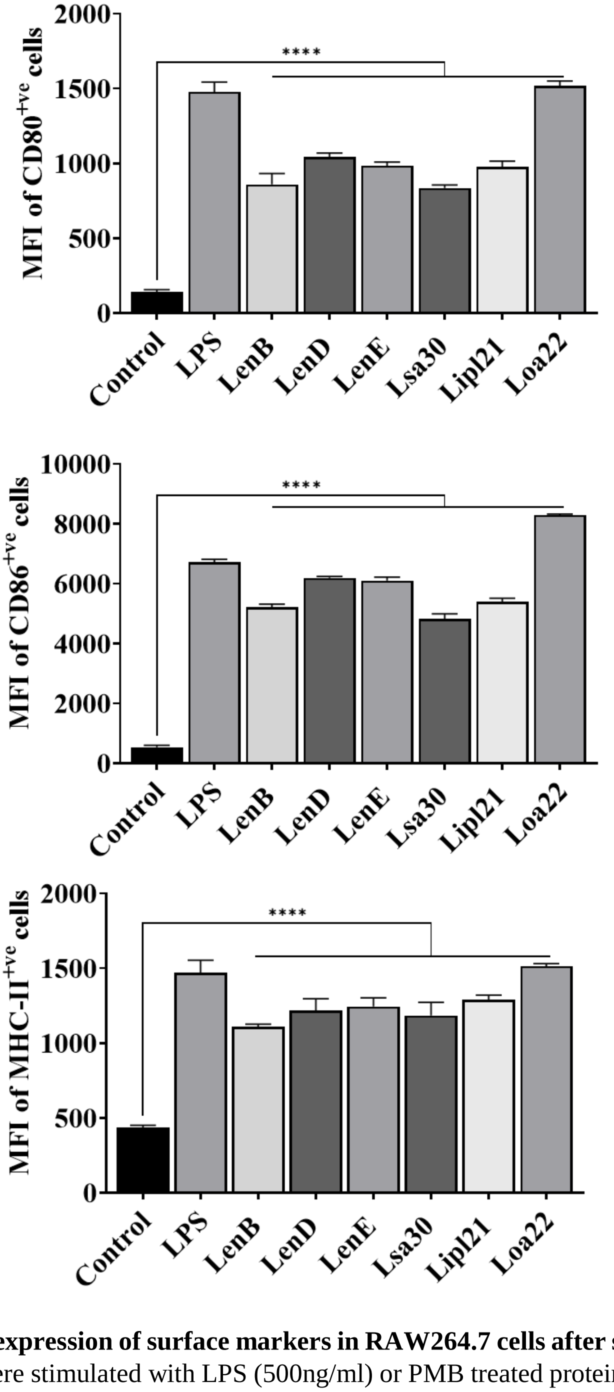

Sup. Fig. 2: Bar graph showing expression of surface markers in RAW264.7 cells after stimulation with surface proteins. RAW264.7 cell lines were stimulated with LPS (500ng/ml) or PMB treated proteins for 24 hrs, followed by stainingwith fluorochrome-conjugated antibodies against CD80, CD86, MHCII and then analysed by Flow cytometry as described in materials and methods. The bar graphs represent the means of mean fluorescence intensity (MFI) ± SEM of positive cells. Data are representative of three independent experiments with duplicate sample in each experiment. Significant differences were calculated using the one-way ANOVA (****indicates $P < 0.0001$)

Sup. Fig. 3

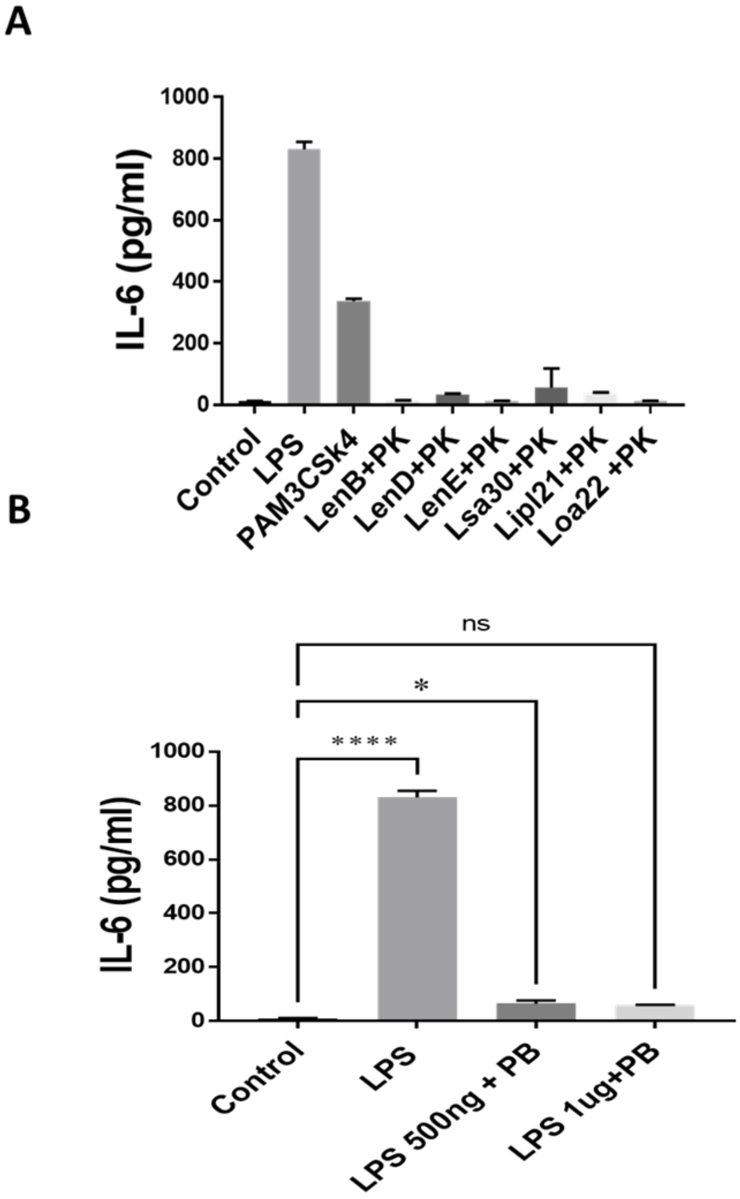

Sup. Fig. 3: Effect of Protease K (PK) and Polymixin B (PMB) on protein and LPS induced production of IL-6 by RAW264.7 cells.(A) *Cytokine induction in RAW264.7 cells by proteins treated with Proteinase K*. The proteins were treated with Proteinase K (5µg/ml protein) at 65°C for 1hr followed by inactivation at 95°C for 5min and then used to stimulate RAW264.7 cells. IL-6 was zanalyzed in culture supernatant by sandwich ELISA kit. **(B)** *Cytokine induction by LPS treated with Polymixin B.* The LPS was treated with Polymyxin B (10µg/ml protein) at 37°C for 1hr and stimulated RAW264.7 cells. IL-6 was analyzed in culture supernatant by sandwich ELISA kit as mentioned in materials and methods. Data are representative of three different experiments. Significant differences were calculated using the one-way ANOVA (****, *, and ns indicates $P < 0.0001$, $P < 0.05$ and non-significant respectively)

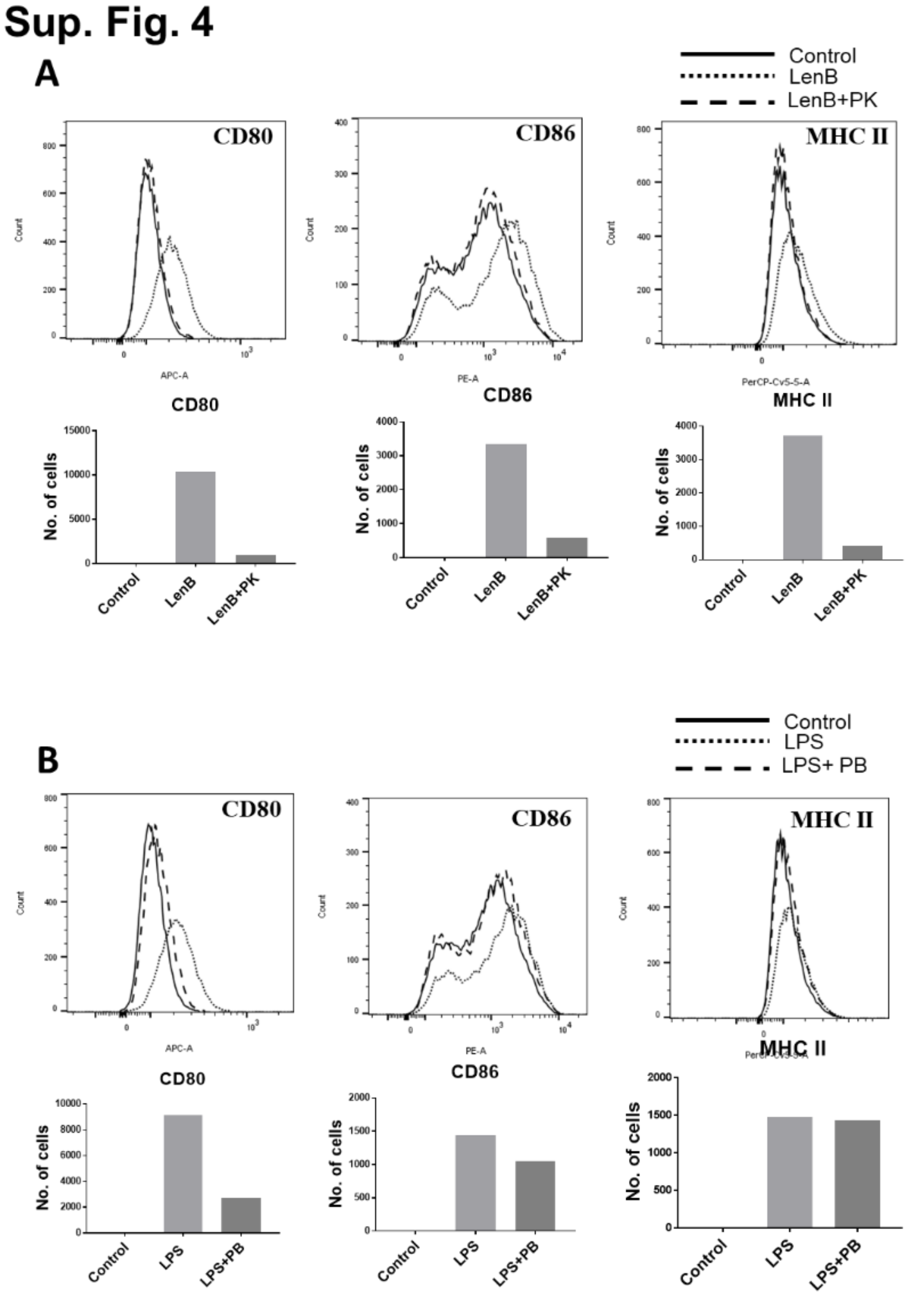

Sup Fig. 4: Effect of Protease K (PK) and Polymixin B (PMB) on protein and LPS induced expression of surface molecules by RAW264.7 cells. (A) *Expression of surface molecules by proteins treated with Proteinase K*. The protein (Len B) was treated with Proteinase K (5μg/mg protein) at 65°C for 1hr followed by inactivation at 95°C for 5min and then used to stimulate RAW264.7 cells for 24 hrs. Expression of surface markers (CD80, CD86, MHCII) was analyzed by Flow cytometryas described in materials and methods. **(B)** *Expression of surface molecules by LPS treated with Polymixin B.* The LPS was treated with Polymyxin B (10μg/ml protein) at 37°C for 1hr and was used to stimulate RAW264.7 cells for 24hrs . The surface markers were analyzed by Flow cytometry as described in materials and methods.

Sup. Fig. 5

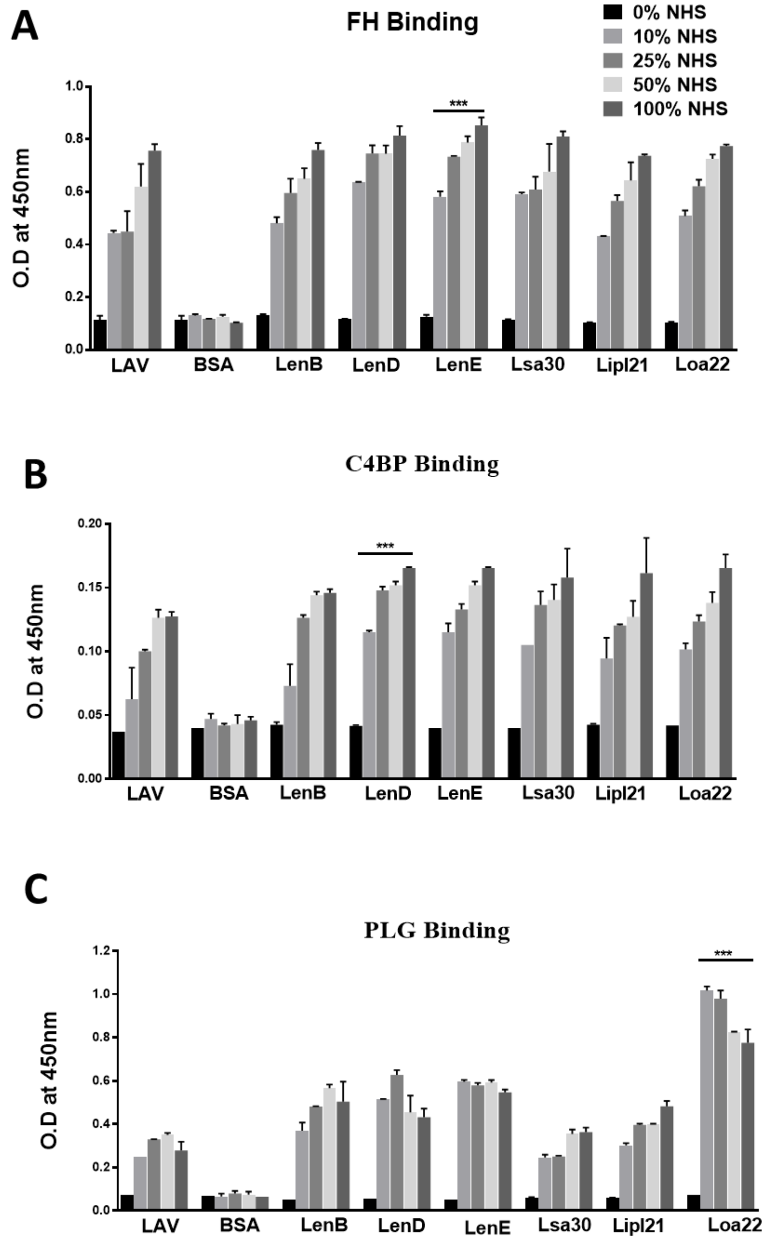

Sup. Fig. 5 Evaluation of binding of surface proteins with FH, C4BP, and PLG in varying concentrations of NHS. Microtitre plates were coated with BSA (negative control), LAV (positive control) or 1µg purified proteins (rLenB or rLenD or rLenE or rLsa30 or rLipl21 or rLoa22) and then incubated with varying concentration of Normal Human Serum (0, 10, 25, 50 and 100%.). The binding was detected with specific antibodies against FH, C4BP or PLG as described in materials and methods. Data are representative of three different experiments. Significant differences were calculated using the two-way ANOVA (*** indicates; $P < 0.001$).
